# Medical Management of Vulvar Vestibulitis: Results of a Sequential Treatment Plan

**DOI:** 10.1155/S1064744995000603

**Published:** 1995

**Authors:** Paul Nyirjesy, Meredith Halpern

**Affiliations:** Department of Obstetrics, Gynecology, and Reproductive Sciences Temple University School of Medicine 3401 N. Broad Street Philadelphia PA 19140 USA

## Abstract

*Objective:* The objectives of this study were to assess the efficacy of medical management for vulvar vestibulitis and to examine several historical variables and determine their predictive values as to which treatments will be most successful.

*Methods:* Seventy-four patients diagnosed with vulvar vestibulitis were evaluated. Each patient was treated using a sequence of consecutive medical therapies for vulvar vestibulitis. These therapies were topical aqueous 4% lidocaine with intercourse, topical corticosteroid therapy, oral amitriptyline, topical low-dose 5-fluorouracil (5-FU) cream, intralesional alpha-interferon, and a low-oxalate diet in combination with oral calcium citrate. The patients were followed over 3–30 months and their responses to therapy were assessed. In addition, a statistical analysis was performed to determine the positive predictive values of certain historical variables and specific treatment successes.

*Results:* Forty-nine patients reported positive responses to one of the initiated therapies. More specifically, 18.1% of the patients who used lidocaine, 33.8% who used topical corticosteroids, 57.1% who used amitriptyline, 16.7% who used 5-FU, none who received interferon, and 50% who tried a low-oxalate diet had positive responses to therapy. No historical variables were predictive of which therapies would have the most successful outcome.

*Conclusions:* Medical management is effective in alleviating the symptoms of vulvar vestibulitis. Various aspects of a patient's history are not helpful in selecting the therapy that will be most effective.


Infectious Diseases in Obstetrics and Gynecology 3:193–197 (1995)
© 1996 Wiley-Liss, Inc.

### Medical Management of Vulvar Vestibulitis: Results of a Sequential Treatment Plan

**Paul Nyirjesy and Meredith Halpern**

*Department of Obstetrics, Gynecology, and Reproductive Sciences, Temple University School of Medicine, Philadelphia, PA*

Abstract

***Objective:*** **The objectives of this study were to assess the efficacy of medical management for vulvar vestibulitis and to examine several historical variables and determine their predictive values as to which treatments will be most successful.**

***Methods:*** **Seventy-four patients diagnosed with vulvar vestibulitis were evaluated. Each patient was treated using a sequence of consecutive medical therapies for vulvar vestibulitis. These therapies were topical aqueous 4% lidocaine with intercourse, topical corticosteroid therapy, oral amitriptyline, topical low-dose 5-fluorouracil (5-FU) cream, intralesional alpha-interferon, and a low-oxalate diet in combination with oral calcium citrate. The patients were followed over 3–30 months and their responses to therapy were assessed. In addition, a statistical analysis was performed to determine the positive predictive values of certain historical variables and specific treatment successes.**

***Results:*** **Forty-nine patients reported positive responses to one of the initiated therapies. More specifically, 18.1% of the patients who used lidocaine, 33.8% who used topical corticosteroids, 57.1% who used amitriptyline, 16.7% who used 5-FU, none who received interferon, and 50% who tried a low-oxalate diet had positive responses to therapy. No historical variables were predictive of which therapies would have the most successful outcome.**

***Conclusions:*** **Medical management is effective in alleviating the symptoms of vulvar vestibulitis. Various aspects of a patient's history are not helpful in selecting the therapy that will be most effective.** © 1996 Wiley-Liss, Inc.

Key words

Amitriptyline, dyspareunia, lidocaine, corticosteroids, low-oxalate diet

Vulvar vestibulitis, a common cause of dyspareunia in women, continues to be of unknown etiology. This entity was first described by Skene[1] in 1889 as "hyperesthesia" or "excessive sensitivity" of the vulvar vestibule. He reported patients with no external manifestations of disease who experienced great pain with any contact with the sensitive areas. In 1891, Thomas and Munde[2] speculated that women with this malady had "an excessive sensibility of the nerves supplying the mucous membranes" of the vulvar vestibule. Kelly[3] also attributed cases of superficial localized dyspareunia to "sensitive deep-red spots in the mucosa of the hymenal ring." For almost 50 years, vulvar hyperesthesia was unmentioned in the medical literature. Peckham and colleagues[4] fully characterized "focal vulvitis," a syndrome in which a patient history of persistent vulvovaginal burning or discomfort is associated with dyspareunia or the insertion of tampons. The diagnostic criteria for this syndrome are as follows: vulvar pain, dyspareunia, or pain with insertion of tampons; one or more minute, exquisitely sensitive, focal inflammatory lesions located in the vestibule; and no identifiable cause such as herpes progenitalis, bacterial or candidal vaginitis, or pemphigoid.

In the past, surgical excision of the vestibule was

Address correspondence/reprint requests to Dr. Paul Nyirjesy, Department of Obstetrics, Gynecology, and Reproductive Sciences, Temple University School of Medicine, 3401 N. Broad Street, Philadelphia, PA 19140.

Received August 29, 1995
Accepted December 19, 1995

the mainstay of therapy. A conservative stepwise approach to vulvar vestibulitis utilizing topical lidocaine and steroids, oral amitriptyline, low-oxalate diet with oral calcium citrate, intralesional interferon, and topical 5-fluorouracil (5-FU) cream was developed as an alternative to what many patients perceived as a radical approach to their disease. The objective of this article is to detail our experience with this approach. We sought to determine whether historical variables such as the duration and type of symptoms, history of documented human papillomavirus (HPV) infection, history of antifungal use, and positive fungal cultures could suggest which therapy would be most likely to be effective.

### SUBJECTS AND METHODS

From a cohort of an estimated 750 patients referred for the evaluation of chronic vaginal symptoms to the Temple University Vaginitis Referral Center, 74 patients diagnosed with vulvar vestibulitis were identified. For each patient, a medical and sexual history was taken with special attention paid to the vaginal symptoms. The quality and severity of symptoms with regard to dyspareunia, burning, itching, swelling, and discharge were scored, and the duration of the symptoms and the patient's age at the onset of her symptoms were recorded. The symptoms were scored in the following manner: absent = 0, minimal = 1, moderate = 2, and severe = 3. For the statistical analysis, scores of 0 or 1 were considered negative, and scores of 2 and 3 were counted as positive.

On the patient's first presentation as well as at subsequent follow-up visits, fungal cultures were obtained from the vagina and vestibule and a microscopic examination of the discharge with saline and 10% potassium hydroxide preparations was performed. The patients with normal microscopic findings who fulfilled the criteria for vulvar vestibulitis were included in this analysis. Any patient with fungal elements present on microscopy or with positive yeast cultures was started on a 6-month suppressive regimen of oral fluconazole, 200 mg weekly. If a non-*Candida albicans* yeast was isolated, a mycologic cure was achieved with boric-acid suppositories. If, 2 months after a sustained mycologic cure, the patient still fulfilled the diagnostic criteria, the treatment for vulvar vestibulitis was initiated.

Each patient was assigned to a sequential treatment plan consisting of one or more of the following therapies: 1) avoidance of any external irritants and the use of aqueous 4% lidocaine applied to the vestibule prior to intercourse for 1 month; 2) topical 0.25% desoximetasone cream applied twice daily to the vestibule for 1 month; 3) oral amitriptyline started at a low dose and gradually increased to the dose that alleviated the symptoms up to a maximum of 100 mg daily for 3 months; 4) topical 5-FU cream applied once weekly to the vestibule for 1 month; 5) injections with recombinant alpha-interferon, 12 injections of 1 million units each in a clockwise fashion to the vestibule over the course of 4 weeks, followed by observation for another 4 weeks; and 6) a low-oxalate diet with calcium citrate supplementation for up to 3 months according to the protocol proposed by Solomons and colleagues.[5] Each patient was followed to assess the tolerance and response to the therapy. A positive response to therapy was recorded if the patient reported an improvement or amelioration of the symptoms to the extent that she was having few symptoms in her daily activities and could have enjoyable intercourse with minimal pain. A negative response was recorded if the therapy was not tolerated because of side effects or if no improvement or worsening of the symptoms was noted. Only one treatment modality was used at any given time.

A statistical analysis using the Epi Info statistical software package (Centers for Disease Control, Atlanta, GA) was carried out in order to assess whether the duration of symptoms; positive yeast cultures; history of HPV infection; prior antifungal use coinciding with the onset of vulvar burning; or dysuria, vaginal discharge, dyspareunia, itching, burning, or swelling was associated with a favorable response to a particular therapy. The categorical data were analyzed for significance by means of the Mantel-Haenszel chi-square formula. If a value of <5 was encountered, a 2-tailed $P$ value was obtained with the Fisher exact test.

### RESULTS

The mean patient age was 30.2 ± 5.9 years. The mean age at the onset of vaginal symptoms was 26.5 ± 6.5 years. The mean duration of symptoms was 42 ± 42.6 months, with a range of 4–190 months. All but one patient were white. Forty-six (62.2%) patients were nulligravid and 61 (82.4%) patients were nulliparous.

The vaginal symptoms and responses to therapy

TABLE 1. Response to various therapies

| Therapy | No. of patients receiving therapy | No. of patients with positive responses to therapy |
|---|---|---|
| Lidocaine | 72 | 13 (18.1%) |
| Desoximetasone | 68 | 23 (33.8%) |
| Amitriptyline | 35 | 20 (57.1%) |
| 5-FU | 6 | 1 (16.7%) |
| Alpha-interferon | 4 | 2 (50%)[a] |
| Low-oxalate diet/calcium citrate | 14 | 7 (50%) |

[a]Vestibulitis recurred in both patients.

were recorded and scored as previously indicated. In 56 (75.7%) patients, the main symptom was dyspareunia. In terms of day-to-day symptoms, 24 (32.4%) patients noted significant itching, 49 (66.1%) burning, 24 (32.4%) swelling, 33 (44.6%) a noticeable vaginal discharge, and 35 (47.3%) dysuria. Three of the 74 (4%) patients had biopsy-proven interstitial cystitis.

Twenty-six (35.1%) patients had undergone vulvar biopsies either by their referring gynecologists or during the course of this study. All of the biopsy specimens reported varying degrees of inflammation. Ten (38.5%) were suggestive of HPV infection; one showed eczematous dermatitis.

By the time the patients with vulvar vestibulitis had been referred to us, 67 (90.5%) had had at least one prior course of topical antimycotic therapy. Of these, 33 (44.6%) reported that the onset of their vaginal burning and dyspareunia coincided with the use of this therapy. Despite repeated fungal cultures at each visit, only 18 (24.3%) had a documented presence of yeast: 15 *C. albicans*, 2 *C. parapsilosis*, and 1 *Saccharomyces cerevisiae.*

The time of follow-up was between 3 and 30 months (mean 18.23 ± 7.0 months). The follow-up was discontinued when a positive response was achieved with a particular mode of therapy and the patient felt that her symptoms had diminished to an acceptable or a nonexistent level. The responses to various therapies are presented in Table 1. Thirteen (18.1%) patients improved with lidocaine and the removal of external irritants; however, 9 of these women decided to attempt a trial of corticosteroids to further improve their symptoms. Of the 20 patients who responded to amitriptyline, 10 required a dose of 75 or 100 mg to respond. Although 2 of the 4 patients receiving interferon had initial positive responses, both recurred with severe vestibulitis within 3 months. These patients were scored as failures. Overall, 49 (66.2%) of our cohort of 74 patients achieved an alleviation of their symptoms. Of the remaining patients, 2 underwent vestibulectomies with the complete resolution of their symptoms. Three were, at their request, referred elsewhere for laser surgery of the vestibule. The remaining 20 patients were lost to follow-up or did not wish to attempt further therapy.

A univariate analysis was used to determine if certain historical factors were predictive of the response to a particular therapy. A positive response to therapy was not correlated with the duration of symptoms, the concomitant presence of yeast, or the presence of dysuria, dyspareunia, vulvar burning or itching, or vaginal discharge.

### DISCUSSION

Vulvar vestibulitis remains a syndrome of unknown etiology. Biopsy specimens taken of the affected areas of the vestibule usually show a nonspecific chronic inflammatory infiltrate in the subepithelial tissues which sometimes involves the ducts of the minor vestibular glands.[4] This finding may represent the endpoint of a number of pathologic processes. The second most common pathologic finding in vestibulitis is epithelial dysplasia with koilocytotic atypia consistent with HPV infection.[6] However, according to Oakes,[7] "There does not appear to be a characteristic histological pattern in focal vulvitis and certainly nothing which gives a clue as to its causation."

The bladder, urethra, and vestibule are all derived from the embryonic urogenital sinus. Therefore, it has been postulated that a urogenital abnormality or autoimmune disorder, with antibodies directed toward the vulvar tissue, may account for vestibulitis[8] as well as interstitial cystitis. However, only 3 of our 74 patients had cystoscopic evidence of interstitial cystitis in addition to vestibulitis.

A history of fungal infection is present in almost all patients who present with vulvar vestibulitis. However, it is impossible to know whether a patient's vestibular symptoms are mistakenly treated as a fungal infection when yeast are not truly present or whether the vestibular symptoms indeed coincide with or follow a real yeast infection. Ashman and Ott[9] proposed that antigens of *C. albicans* are cross-reactive with certain vulvovaginal tissue antigens which may account for the high association of vestibulitis with a previous yeast infection. However, according to Marinoff and Turner,[10] it is unlikely that a delayed hypersensitivity reaction to *Candida* or topical antifungal preparations is the cause of vestibulitis. Despite these findings, it is difficult to ignore the high incidence of vestibulitis in women who associate the onset of their symptoms with the use of antifungals. Similarly, the relatively high (24.3%) incidence of coexisting fungal vaginitis in our patient population suggests that recurrent yeast infections may somehow play a role in the initiation of this syndrome.

It has been postulated recently by Solomons et al.[5] that the pattern of pain associated with vestibulitis is related to urinary pH and urinary oxalate excretion. Increased pain may be associated with urinary pH values of 6.5–8.5 and transient hyperoxaluria which can cause burning on contact with oxalate crystals or solution on vulvar mucosa. A low-oxalate diet and calcium citrate supplementation are thought to retard the nucleation and crystal growth of calcium salts and decrease vestibular pain. Of the patients who attempted this therapy, 50% noted significant improvement. Interestingly, 3 in the failure group stopped the therapy because they were unable to tolerate either the diet or the calcium citrate pills.

HPV has been suspected as a possible cause of the vulvar vestibulitis syndrome. Turner and Marinoff[11] studied 7 patients with vestibulitis who were positive for HPV by DNA hybridization techniques. The biopsies of these patients failed to show HPV changes in all cases. They postulated that an inflammatory response around the superficial capillaries may be the beginning of the body's attempt to rid itself of the virus. In later stages, there is dense lymphocytic infiltration so that the typical histologic features of HPV may not be seen. The antiviral and immunomodulatory action of alpha-interferon is thought to be the mode of action in the treatment of vestibulitis. The intralesional injection of recombinant alpha-interferon has been used in some studies with limited success. Marinoff et al.[12] found that only 18% of the women they treated with interferon showed substantial improvement (46/55 women treated had HPV changes by vulvar biopsy) and the response to interferon was not influenced by the diagnosis of HPV. Our initial experience with interferon therapy was also disappointing, and the discomfort associated with this therapy led to our discontinuation of its use.

Among the more successful treatment modalities has been the use of amitriptyline (or other tricyclic antidepressants). This modality has been used in the treatment of vulvodynia in which it is felt to target the potential neurologic component of vulvar pain and burning.[13] This neurologic component is postulated to be a problem with cutaneous perception, either centrally or at the nerve root. Tricyclic antidepressants have been used successfully to treat glossodynia and postherpetic neuralgia, 2 forms of cutaneous dysesthesia of which vulvar vestibulitis may also be a member. Amitriptyline inhibits the membrane pump mechanism responsible for the uptake of norepinephrine and serotonin in adrenergic and serotonergic neurons which may potentiate or prolong neuronal activity. This underlying mechanism is believed to be responsible for the antidepressant activity, although its true antidepressant mechanism is unknown. In vulvodynia patients, McKay[13] found the results of treatment with amitriptyline to be most successful in older women. The success in treating vulvar vestibulitis with amitriptyline may be attributed to its effect on cutaneous nerve as well as its psychotropic effect. However, antidepressants have been shown to be effective in treating many chronic pain syndromes with or without the presence of depression. In this study amitriptyline was the most successful medical therapy, with a 55.9% response. The addition of biofeedback therapy to this regimen may lead to even better results.[14]

Surgical excision of the vestibule has been met with some success in the treatment of vestibulitis. Mann and colleagues[15] reported a 66% success rate and regression of symptoms following surgical excision by perineoplasty. However, concerns about the cost, risks, and potential scarring frighten many women away from seeking therapy for their disease. With an overall success rate of 66.2% in our study, medical management offers an attractive alternative to surgery. However, the relatively high number of patients who were lost to follow-up or did not return for further treatment underscores the downside of a stepwise approach to management, namely the need for more frequent office visits and what patients may perceive as very slow progress.

Vulvar vestibulitis is a chronic syndrome that seems to respond to different therapies. In the present study of 74 patients with vulvar vestibulitis, a conservative medical approach was taken using the previously mentioned modalities. The varying effectiveness of these regimens supports the use of a stepwise protocol, starting with simple treatments with few side effects. With such a protocol, many women with vulvar vestibulitis can achieve adequate relief of their symptoms with medical treatment alone.

### REFERENCES

1. Skene AJC: Treatise on the Diseases of Women. New York: Appleton & Company, 1889.
2. Thomas TG, Munde PF: Hyperaesthesia of the vulva. In: The Diseases of Women. Philadelphia: Lea Brothers & Co., 1891.
3. Kelly HA: Gynecology. New York: Appleton & Company, p 236, 1928.
4. Peckham B, Maki D, Patterson J, Gholan-Reza H: Focal vulvitis: A characteristic syndrome and cause of dyspareunia. Am J Obstet Gynecol 154:855–864, 1986.
5. Solomons LL, Melmed MH, Heitler SM: Calcium citrate for vulvar vestibulitis. J Reprod Med 36:879–882, 1991.
6. Furlonge CB, Thin RN, Evans BE, McKee PH: Vulvar vestibulitis syndrome: A clinico-pathological study. Br J Obstet Gynaecol 98:703–706, 1991.
7. Oakes JK: Focal vulvitis and localized dyspareunia. Genitourin Med 66:28–30, 1990.
8. McCormack W: Two urogenital sinus syndromes: Interstitial cystitis and focal vulvitis. J Reprod Med 38:873–876, 1990.
9. Ashman RB, Ott AK: Autoimmunity as a factor in recurrent vaginal candidiasis and the minor vestibular gland syndrome. J Reprod Med 34:264–266, 1989.
10. Marinoff S, Turner M: Hypersensitivity of vaginal candidiasis or treatment vehicles in the pathogenesis of minor vestibular gland syndrome. J Reprod Med 31:796–799, 1986.
11. Turner M, Marinoff S: Association of human papillomavirus with vulvodynia and the vulvar vestibulitis syndrome. J Reprod Med 33:533–537, 1988.
12. Marinoff S, Turner M, Hirsch R, Richard G: Intralesional alpha interferon: Cost-effective therapy for vulvar vestibulitis syndrome. J Reprod Med 38:19–24, 1991.
13. McKay M: Dysesthetic vulvodynia: Treatment with amitriptyline. J Reprod Med 38:9–13, 1993.
14. Glazer HI, Rodke G, Swencionis C, Hertz R, Young AW: Treatment of vulvar vestibulitis syndrome with electromyographic biofeedback of pelvic floor musculature. J Reprod Med 40:283–290, 1995.
15. Mann M, Kaufman R, Brown D, Adam E: Vulvar vestibulitis: Significant clinical variables and treatment outcomes. Obstet Gynecol 79:122–125, 1992.
